# Myopia Correction, Myopia Control and Myopia Management: Definitions and Recommended Usage

**DOI:** 10.1167/iovs.66.6.41

**Published:** 2025-06-12

**Authors:** Ian Flitcroft, Mark A. Bullimore, Kate L. Gifford, Jost B. Jonas, Deborah Jones, Lyndon W. Jones, Pauline Kang, Serge Resnikoff, Jeffrey Walline, Christine F. Wildsoet, On behalf of the International Myopia Institute (IMI)

**Affiliations:** 1 Centre for Eye Research Ireland, Sustainability and Health Research Hub, Technological University Dublin, Dublin, Ireland; 2 College of Optometry, University of Houston, Houston, Texas, United States; 3 School of Optometry and Vision Science, Centre for Vision and Eye Research, Faculty of Health, Queensland University of Technology, Australia; 4 Rothschild Foundation Hospital, Institut Français de Myopie, Paris, France; 5 School of Optometry & Vision Science, University of Waterloo, Waterloo, Ontario, Canada; 6 School of Optometry and Vision Science, UNSW, Sydney, Australia; 7 College of Optometry, The Ohio State University, Columbus, Ohio, United States; 8 School of Optometry, University of California, Berkeley, Berkeley, California, United States

The terms “myopia management” and “myopia control” are frequently used in both eye care and research settings, sometimes interchangeably, leading to potential confusion. Although both terms are important and useful in different situations, they represent fundamentally distinct concepts. When using such terms, it is important to consider the context and application—for example, whether they are being discussed with parents or included in written materials such as medical publications, marketing content, or regulatory documents. Their use may also vary by country. The aim of this article is to provide clear definitions of these terms, based on their evolution in recent years,^1^^–^^4^ and informed by consultations with industry representatives and members of the myopia research community.

In relation to discussions with parents, the most important distinction is not between myopia management and myopia control, but between the concept of active management of myopia as compared to simple vision correction. The recent National Academies of Science Engineering and Medicine (NASEM) report often uses the phrase “traditional myopia management” to mean “single vision correction,” which could be confusing to parents presented with two options, “traditional management” and “active management.”^5^ To ensure parents easily grasp the distinction between these approaches to management, the term “myopia correction” is recommended to describe the traditional approach.

**Myopia Correction:** Devices and interventions that correct the optical focusing errors of myopia to optimize best corrected distance visual acuity, without providing any intended benefits in relation to slowing myopia progression or axial elongation.

“Myopia management” should be reserved for approaches that combine myopia correction with a comprehensive approach to eye and vision care that aims to also slow myopia progression and reduce the risk of future, myopia-associated complications. Myopia management represents an evidence-based approach to eye and vision care for both patients with myopia and those at risk of developing it (i.e., pre-myopia).^6^^,^^7^ It encompasses myopia risk assessment, early detection through regular vision screening, appropriate optical correction, and timely interventions to prevent and control myopia progression and excessive axial elongation. Myopia management also includes lifestyle recommendations, such as increased outdoor time and reduced screen exposure, and ongoing monitoring of refraction and axial length.^8^^,^^9^ Importantly, myopia management should also address the long-term care and treatment of emerging myopia-related complications across the entire lifespan of the patient.^10^

**Myopia Management:** Myopia management is a comprehensive eye and vision care approach to myopia and pre-myopia that includes prevention, myopia risk assessment, early detection through screening, appropriate correction, lifestyle recommendations, interventions to reduce myopia progression and axial elongation, monitoring of refraction and axial length, and management of emerging myopia-related complications.

Myopia control typically refers to the use of interventions specifically aimed at slowing down progression of myopia and reducing associated risks of sight-threatening myopic complications and, in this sense, is a narrower, more specific term. Such interventions may serve dual roles, also providing optical correction, or may only address the refractive progression and excessive axial elongation. In the latter case, the myopia treatment may need to be combined with myopia correction. Monitoring of treatment efficacy with refraction and axial length measurements is an integral part of myopia control.^11^ In addition, there is also a very important regulatory dimension to myopia control in many countries.^3^ In many jurisdictions, such as the USA, myopia control has a specific regulatory meaning, with approved products that have as part of their indications for use, “myopia control” or that refer to “slowing the progression of myopia,” having demonstrated sufficient clinical evidence of efficacy to justify regulatory approval.

Regulatory approval of a product need not indicate that its efficacy in reducing myopia progression has been evaluated. Confusingly, there are products used for myopia control that have been given marketing clearance for “myopia management” but not “myopia control.” This distinction reflects the fact that their efficacy in slowing myopia progression or axial elongation was not assessed as part of the regulatory clearance process. In Europe, the CE marking that denotes an approved product does not specifically differentiate between spectacles used for myopia control (slowing myopia progression) and standard spectacles used for simple myopia correction (i.e., optical optimization of distant visual acuity). Both types of spectacles, regardless of therapeutic claims regarding myopia control, must meet the same fundamental CE marking standards for materials, manufacturing, and safety, based on materials and manufacturing standards (such as EN ISO 14889, EN ISO 21987, and ISO 8980). An optical product designed for myopia control can therefore be approved in Europe without needing to prove efficacy, provided no medical claims regarding the latter are made.

**Myopia Control:** The clinical application of evidence-based interventions specifically intended to slow the progression of myopia and axial elongation. Such interventions may also correct refractive errors (i.e., provide “myopia correction”). Myopia control constitutes one essential component within the broader scope of myopia management and should include monitoring of treatment efficacy. When describing a specific product in clinical communications, the term “myopia control” should only be applied to evidence-based interventions supported by clinical trials indicating efficacy in slowing myopia progression and/or axial elongation. Evidence for efficacy should include cycloplegic refraction and axial length data. For some interventions, such as orthokeratology, axial length alone is appropriate. Refraction, in isolation, is not adequate evidence of efficacy. When used in product marketing, this should also be supported by regulatory approval.

The diagram shown in the Figure demonstrates the relationship between myopia correction, myopia control, and myopia management. Myopia control, myopia correction, and the monitoring of refraction and axial length are the core clinical elements of myopia management. The concept of myopia management also encompasses the need for prevention, education, communication, risk assessment, vision screening, lifestyle recommendations, and the monitoring/management of potential myopia-related complications. Although clinicians may participate in many of these activities, a wide range of stakeholders needs to be involved to provide optimal myopia management at a public health level.

**Figure.**
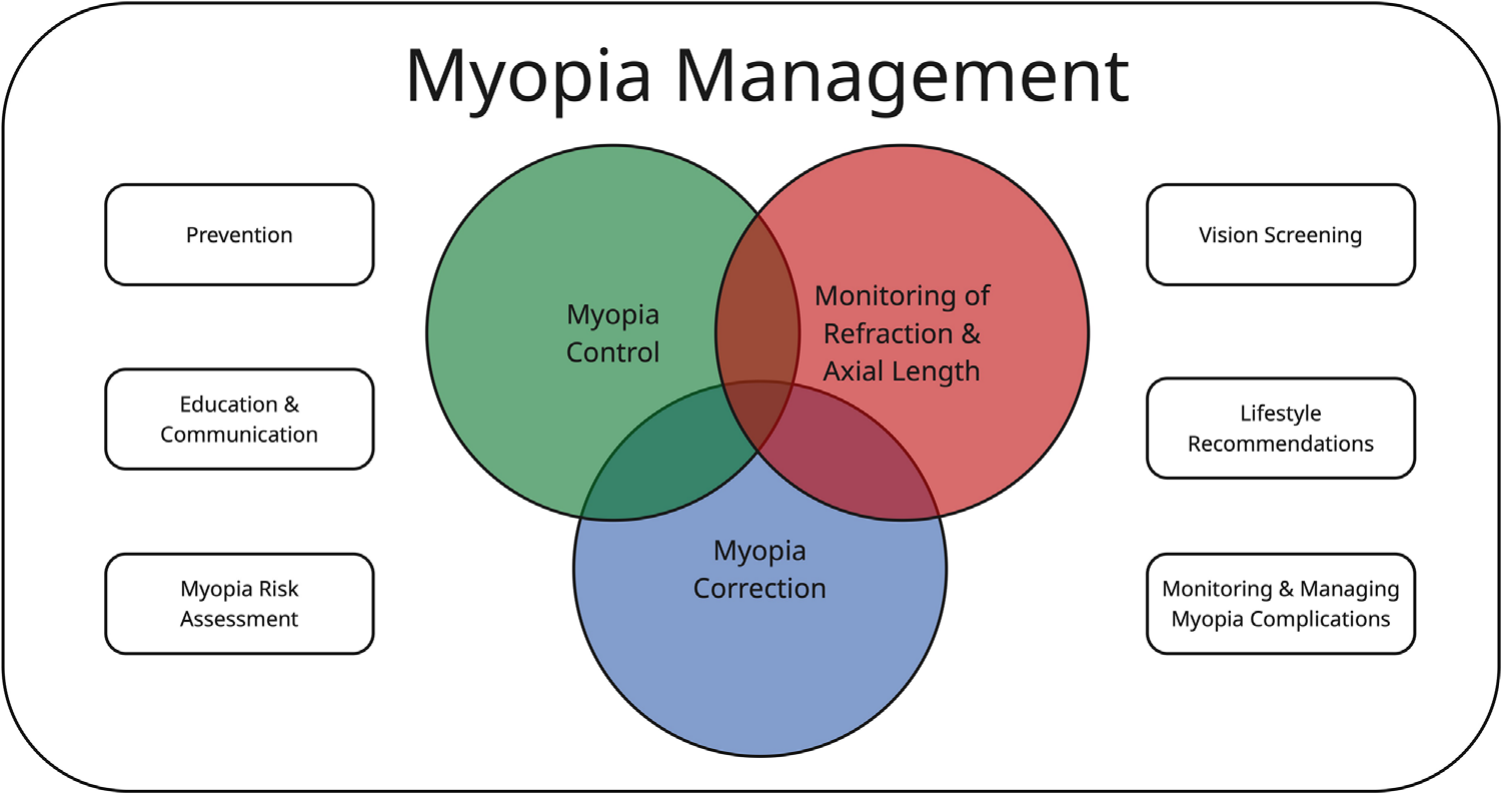
Representation of the relationship among myopia management, myopia control, and myopia correction, and other important aspects of myopia management.

Attention has also been drawn to the question of whether the term “myopia control” carries with it, the implication that myopia can be fully controlled (i.e., progression arrested). To avoid falsely raising the expectations of parents and, potentially, also of clinicians, the alternative phrase “myopia progression management” has been proposed. However, introducing another term at this stage is more likely to increase confusion than improve clarity. Furthermore, in many other medical fields, the word “control” is used without any implication that treatment is always fully successful, for example when used in relation to glucose control and blood pressure control.

## Summary

In relation to the overall concept of active myopia management, the term “Myopia Management” should be the preferred term used in communication with parents and patients. It is also the appropriate term to use in clinical guidelines, as it represents a comprehensive, clinical approach to myopia. “Myopia Correction” can be used to describe the provision of optical correction, with emphasis that while this is an integral component of myopia management, it does *not* represent an adequate level of care by itself in someone demonstrating, or at risk of, myopic progression. The term “myopia control” should be used in a clinical or research context for evidence-based interventions proven to slow myopia progression and/or axial elongation. In marketing and promotional materials, its use should be reserved for evidence-based products supported by clinical trials that demonstrate slowing of myopia progression or axial elongation and accompanied by appropriate regulatory approval/certification. Evidence for efficacy should include cycloplegic refraction and axial length data. For some interventions, such as orthokeratology, axial length alone is appropriate. Refraction, in isolation, is not adequate evidence of efficacy.
